# Rationale and design of REGULATE: an observational study protocol for relationship between plasma metabolome and the efficacy of systemic glucocorticoid in acute exacerbation of chronic obstructive pulmonary disease

**DOI:** 10.1186/s12890-021-01614-3

**Published:** 2021-07-28

**Authors:** Qiu-Yu Li, Zhuo-Yu An, Zi-Han Pan, Rui-Ying Qi

**Affiliations:** 1 Department of Respiratory and Critical Care Medicine, Peking University Third Hospital, Beijing, 100191 People’s Republic of China; 2 Peking University Institute of Hematology, Peking University People’s Hospital, Beijing, 100191 People’s Republic of China

## Abstract

**Background:**

Acute exacerbation of chronic obstructive pulmonary disease (AECOPD) significantly increases the mortality of patients with COPD. Guidelines have recommended systemic glucocorticoid as a regular treatment. Recently, evidence has shown that systemic glucocorticoid cannot be a benefit to all of the patients with AECOPD. Thus, the problem that how the clinicians can screen the patients who can benefit from systemic glucocorticoid needs to be solved urgently. This study is aimed to detect the metabolic biomarkers and metabolic pathways that are related to the efficacy of systemic glucocorticoid and contribute to the precise treatment of COPD.

**Methods and design:**

In this study, we will utilize ultraperformance liquid chromatography/mass spectrometry (LC–MS) and gas chromatography/mass spectrometry (GC–MS) methods for the analysis of the metabolites in AECOPD patients and compare the metabolites profiles between patients with systemic glucocorticoid treatment success group and treatment failure group. We aim to detect the metabolic biomarkers and metabolic pathways that are related to the efficacy of systemic glucocorticoid and contribute to the precise treatment of COPD.

**Discussion:**

Previous studies have found that plasma metabolome changed significantly after dexamethasone treatment in healthy participants. Furthermore, inter-person variability was high and remained uninfluenced by treatment, suggesting the potential of metabolomics for predicting the efficacy and side effects of systemic glucocorticoid. Therefore, we hypothesized that metabolome changes in patients with AECOPD may be associated with the efficacy of systemic glucocorticoid.

*Trial registration* Clinicaltrials.gov registration number NCT04710849. Registered 15 January 2021, https://clinicaltrials.gov/ct2/show/NCT04710849.

## Background

### Disease background and relevant pathobiology/epidemiology that the proposed study addresses

COPD is a common chronic respiratory disease that seriously endangers human health. At present, according to the World Health Organization, there are about 300 million COPD patients worldwide, and COPD has become the fourth cause of death in the world [1]. The global consumption of drugs for COPD reaches more than US$44 billion dollars annually [2]. However, existing therapies have not really altered the prognosis of COPD, especially for AECOPD, which significantly decreases the quality of life and increases mortality [1, 3, 4].

Systemic glucocorticoid therapy can shorten the course of acute exacerbation of COPD, improve lung function and arterial blood oxygen, reduce the risk of treatment failure, and shorten the length of stay [5, 6]. Therefore, the current guidelines recommend systemic glucocorticoids as one of the conventional treatment drugs for acute exacerbation of lung obstruction [5, 6]. However, in recent years, some scholars have questioned the therapeutic value of systemic glucocorticoid therapy in the acute exacerbation of COPD, and there may be treatment-related risks [7]. The results of multiple studies have shown that systemic glucocorticoid therapy does not benefit all patients with acute exacerbation of COPD, and the failure rate of glucocorticoid therapy is 14.5–39%, which is mainly manifested as persistent non-relief or aggravation of disease symptoms [8–10]. In patients with acute exacerbation of COPD who require mechanical ventilation, systemic glucocorticoid therapy fails to improve the mortality rate and shorten the length of stay in the ICU. On the contrary, it also increases the risk of diabetes [11], which may be caused by glucocorticoids. The catabolism of skeletal muscle is strong, which in turn leads to a decrease in respiratory muscle volume and weakened respiratory muscle strength [12].

Metabolomics mainly uses high-throughput detection techniques such as nuclear magnetic resonance, liquid chromatography (LC), or gas chromatography (GC) combined with mass spectrometry (MS) to qualitatively and quantitatively study the pathophysiological stimulation or genetic genes of living organisms [13]. Metabolomics is mainly concerned with the dynamic changes of various small molecular metabolites, which can reflect the functional state of cells more truthfully and sensitively [14, 15]. In the past ten years, metabolomics has been gradually applied to many research fields such as the pathogenesis of COPD, disease diagnosis, and disease phenotype.

It has been found that metabolomics has a good application prospect for predicting glucocorticoid response and side effects. Bordage et al. compared the administration of dexamethasone in healthy subjects The dynamic changes of plasma metabolite profile before and after a single administration of 4 mg showed that a variety of plasma metabolites changed significantly after administration of dexamethasone, and there were significant differences between individuals and were not affected by treatment [16]. According to changes in metabolites over time and individual differences, it may help to detect serious adverse effects of glucocorticoids early, optimize individual treatment plans, and reduce medical expenditures.

Therefore, based on the current research status at home and abroad and the foundation of the previous research work of this research group, we propose the hypothesis of this research: in patients with acute exacerbation of COPD, the individual differences in the profile of metabolic markers may be related to the success rate of systemic glucocorticoid therapy and the recent It is related to long-term curative effect, combined with metabonomic analysis and some characteristic clinical data to construct a predictive model, which may help to screen outpatients with acute exacerbation of COPD who respond well to systemic glucocorticoid therapy, and guide the treatment of acute exacerbation of COPD.

This research group will use the established high-performance liquid chromatography-quadrupole time-of-flight-mass spectrometry (UPLC–QTOF–MS) and gas chromatography-mass spectrometry (GC–MS) analysis methods to apply to patients receiving systemic glucocorticoid therapy Serum metabonomics analysis of patients with acute exacerbation of the COPD, comparing the differences in serum metabolic marker profiles between the successful glucocorticoid treatment group and the glucocorticoid treatment failure group, and through multivariate data statistical analysis, metabolite database retrieval, standard comparison and other methods, Screen out possible metabolic markers related to the outcome of systemic glucocorticoid therapy, and further combine with characteristic clinical data to construct a mathematical model to predict the effect of systemic glucocorticoid therapy.

## Methods

### Design, participants, and timeframe of enrollment and visits

#### Study design

The design of this study is a prospective observational cohort study.

#### Recruitment and power

From Feb 2021 to Feb 2024, outpatients and inpatients diagnosed with AECOPD admitted to the department of respiratory and critical care medicine, Peking University Third Hospital will be enrolled. For patients who are admitted multiple times during the study period, only the first admission was included. The inclusion criteria were: acute worsening of respiratory symptoms that results in additional therapy. The exclusion criteria are as follows. (1) Other airflow obstructive diseases such as bronchial asthma and bronchiectasis; (2) combined with community-acquired pneumonia (CAP), hospital-acquired pneumonia (HAP) or aspiration pneumonia; (3) combined with severe liver and kidney insufficiency; (4) malignant tumor; (5) immune deficiency due to chemotherapy or HIV infection; (6) received systemic glucocorticoid therapy for acute exacerbation of COPD within 1 month before this admission; (7) severe trauma or stress, etc. [6]. All participants were given written informed consent.

### Exam/visit components, measurements of exposure and disease, and other measurements

#### Treatment plan

All selected patients were given methylprednisolone 40 mg/d intravenous infusion for continuous treatment for 5–7 days by the treatment plan for acute exacerbation of COPD recommended by GOLD 2020 guidelines; all patients were given short-acting bronchodilator inhalation therapy; antibiotic use Indications for: increased dyspnea, increased sputum volume and purulent sputum; or two necessary symptoms including increased purulent sputum; or the need for invasive or non-invasive mechanical ventilation. The course of antibiotics is 5–10 days.

If the patient has COPD comorbidities such as hypertension, cardiovascular disease, diabetes, etc., maintain the original drug treatment plan.

#### Specimen collection and processing

Before receiving systemic glucocorticoid therapy, all selected patients should retain serum and induced sputum specimens for proper treatment. The blood specimens and induced sputum specimens are additionally tested through established methods.

#### Patient grouping and sample size

**Fig. 1:**
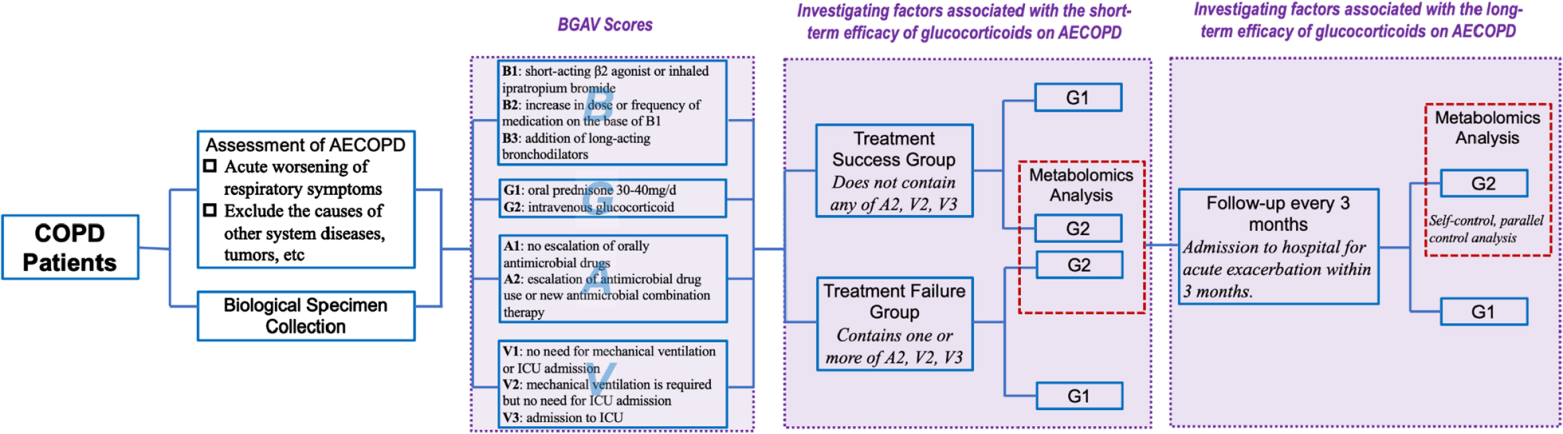
Flow chart of REGULATE study. AECOPD: acute exacerbation of chronic obstructive pulmonary disease; BGAV: a scoring system for AECOPD patients, B: bronchodilator; G: glucocorticoid; A: antibiotic; V: ventilation;

According to the response of systemic glucocorticoid therapy, patients were divided into a treatment success group and treatment failure group. According to clinical experience and previous studies [10], this study established a BGAV scoring system to judge AECOPD treatment success group or treatment failure group (Table 1). The patients whose BGAV score contains one or more of A2, V2, V3 are assigned to the treatment failure group, and the patients who do not contain any of A2, V2, V3 are assigned to the treatment success group. After grouping in this study, metabolomics analysis of patients in the two groups of intravenous glucocorticoids, or G2 group (Fig. 1, Table 2).

**Table1:** Illustration of BGAV scoring system

Term	Grade	Definition
B: bronchodilator	B1	Patients need short-acting β2 receptor agonists or nebulized ipratropium bromide
	B2	The patient's dosage or frequency of β2 receptor agonists on the basis of B1 increased
	B3	Patients need to use long-acting bronchodilators on the basis of B2
G: glucocorticoid	G1	Patients need oral prednisone 30–40 mg/d
	G2	The patient needs intravenous drip of glucocorticoid
A: antibiotics	A1	The infection can be controlled by oral application of firstline antibacterial drugs, without upgrading antibiotic drugs
	A2	The patient's use of antibacterial drugs in this course of treatment is upgraded or new antibacterial drug combination therapy is added
V: ventilation	V1	The patient does not need mechanical ventilation and does not need to be admitted to the ICU
	V2	The patient does not need mechanical ventilation and does not need to be admitted to the ICU
	V3	The patient is admitted to the ICU

**Table 2:** Trial process chart

Period	Run-in period	Treatment period	Follow-up period
Week	− 1	0 to discharge	12, 24, 36, 48 weeks after discharge
Informed consent	√		
Inclusion/exclusion criteria	√		
*Examination*			
Medical examination	√		√
Blood test	√		√
Liver function test	√		
Electrocardiogram	√		
Pulmonary function tests	√		√
mMRC and CAT scoring	√		√
Evaluation of glucocorticoids adverse effects			√
*Treatment and BGAV scoring*			
Bronchodilators		√	√
Glucocorticoids		√	√
Antibiotics		√	
Ventilation		√	
Blood specimens for metabolomics analysis	√	√	√

We use the software PASS 2021 to estimate the sample size. The proportion of successful treatment groups is set to 70%, the proportion of treatment failure group is set to 30%, and the power of the true negative rate of research results is set to 80%. The total sample size required is 117 cases, including 82 cases in the successful treatment group and 35 cases in the treatment failure group.

To facilitate clinical implementation, this study plans to enroll 115–125 patients with acute exacerbation of the COPD, including 80–85 cases in the successful treatment group and 35–40 cases in the treatment failure group.

## Metabolomics analysis and comparison

1. Use UPLC–QTOF–MS AB6600 and GC–TOF–MS Pegasus HT (LECO USA)
  The detection platform detects the endogenous metabolite groups in the serum of all selected patients.
2. Use principal component analysis (PCA), partial least squares discriminant analysis (PLS-DA), orthogonal partial least squares-discriminant analysis (OPLS-DA), and other data analysis methods to screen and determine the differences between the two groups of patients Metabolism markers, and qualitative metabolic markers by searching the self-built reference material database and METLIN public database. The ROC curve was constructed to evaluate the significance of the selected metabolic markers in predicting the response to glucocorticoid therapy.
3. Metabolic pathway analysis: Design a program to classify metabolites into corresponding metabolic pathways, and automatically obtain metabolic pathways based on the results of the 2-step analysis.

## Follow up

The patients were followed up every three months after discharge, and the follow-up period is 1 year. Short-term efficacy indicators include the length of this hospital stay, the time from discharge to the next acute exacerbation, and the side effects of glucocorticoid therapy. Long-term efficacy indicators include mortality within 1 year (overall mortality, COPD-related mortality), frequency of acute exacerbations, frequency of severe acute exacerbations (requiring hospitalization), mMRC, CAT, and changes in lung function (Fig. 1, Table 2).

### Quality assurance and control plans

The research group will set up a Quality Control and Quality Assurance Committee, which will hold an online video conference every three months to review the progress of the trial, the overall result rate, and technical issues.

In the non-targeted metabonomics analysis of plasma by high performance liquid chromatography mass spectrometry (LC–MS), the prepared quality control (QC) samples will be inserted into the analysis samples in the order of arranging one QC sample for every 10 analysis samples, which will be used to monitor the quality control of the analysis samples from sample pretreatment to analysis and detection in real time. The original metabolic fingerprints were analyzed by MS-dial. The relative standard deriviation (RSD) of each metabolite in QC samples was calculated after software pretreatment, which will be controlled below 30%. This will indicate that the quality control of samples from sample pretreatment to analysis and detection was good, and the obtained metabonomics data were true and reliable.

### Data management plan

The clinical data management (CDM) designed an electronic case report form (e-CRF) for clinical data collection (Supplement). The CDM team reviews data each month to ensure that data problems are resolved in a timely manner. Finally, after the completion of all data management activities, a COPD Metabolomics Database will be integrated to further improvement and analysis of the data.

### Analysis plan

All data were analyzed by IBM SPSS statistics 24 and GraphPad prism 7.0 software. Data of normal distribution were presented with mean ± standard deviation (mean ± SD). The mean comparison between the two groups was conducted with Student's t test, and the enumeration data were expressed by rate and analyzed by chi-square test. The rank sum test was used to determine the difference between level data. Independent risk factors are analyzed by multivariable logistic regression. In addition, the Hosmer–Lemeshow test was used for calibration and discrimination was assessed using AUROC. *P* value less than 0.05 was considered to be statistically significant.

## Discussion

AECOPD has obvious heterogeneity in clinical manifestations, prognosis, and treatment response. While following the guidelines, precise treatment and individualized treatment are getting more and more attention. Although systemic glucocorticoid therapy is currently the first-line treatment of AECOPD, it does not benefit all patients, and how to predict which patients will benefit from it is an urgent clinical problem [10, 11, 15, 17, 18].

For example, diabetes, hypertension, and cardiovascular diseases are not only common complications of COPD but also related to AECOPD; our previous study also found that the serum metabolic marker profile of COPD patients is similar to that of the control group. Significant differences. On the other hand, the plasma metabolic profile of healthy subjects changed significantly after receiving glucocorticoid therapy, and the differences between individuals were significant and not affected by the treatment, suggesting that metabolomics can help predict glucocorticoid responses and side effects. Combining previous research results and the previous work of this research group, we propose a research hypothesis: the individual differences in the profile of metabolic markers in AECOPD may be related to the efficacy of systemic glucocorticoids. Therefore, the scientific issues discussed in this study have important academic significance and clinical application value; the basis for the project is sufficient, and the research plan is reasonable. It can screen out AECOPD that can benefit from systemic glucocorticoid therapy, and increase the number of patients the understanding of systemic metabolic control pathways in the acute exacerbation of pulmonary obstruction provides a theoretical basis for the establishment of precise treatment strategies for AECOPD [3, 5, 9, 12].

The treatment of AECOPD follows the recommendations of the current guidelines, which is easy to standardize, reduces the influence of treatment-related confounding factors, and does not violate ethical principles. Secondly, the first sample collection of this study before the application of systemic glucocorticoid therapy can also exclude the effect of glucocorticoid therapy on the body's metabolism.

This research plan is innovative in thought and technology. This project closely combines clinical practice and actual needs. At present, there is still a lack of clinical biomarkers to predict the efficacy of COPD systemic glucocorticoids, and in the period of AECOPD, the changes in systemic metabolic pathways and network regulation mechanisms are still unclear. Through this study, it is possible to find new biomarkers that may be used to predict the efficacy of systemic glucocorticoids, and also help to discover the pathophysiological mechanism of AECOPD from the metabolic level. This study intends to adopt metabonomics methods such as liquid chromatography-mass spectrometry and gas chromatography–mass spectrometry to analyze the profile of metabolic markers and differences between groups in AECOPD, which is more comprehensive, efficient, and accurate [4, 7, 19].

In conclusion, this observational study will compare the metabolomic differences in different effects of glucocorticoid treatment in patients with AECOPD and explore the underlying metabolic pathway mechanisms. The results of this study will provide metabolic markers and metabolic regulatory pathways associated with systemic glucocorticoid efficacy and provide a theoretical basis for precise treatment strategies for AECOPD.

## Data Availability

The data supporting the results of this study will be available upon request. Please contact Dr. Zhuo-Yu An at anzhuoyu@pku.edu.cn to get data.

## Abbreviations

AECOPD: Acute exacerbation of chronic obstructive pulmonary disease
BGAV: A scoring system for AECOPD patients
B: Bronchodilator
G: Glucocorticoid
A: Antibiotic
V: Ventilation
CAP: Community-acquired pneumonia
CAT: COPD assessment test
CDM: Clinical data management
COPD: Chronic obstructive pulmonary disease
e-CRF: Electronic case report form
GC: Gas chromatography
HAP: Hospital-acquired pneumonia
HIV: Human immunodeficiency virus
ICU: Intensive care unit
LC: Liquid chromatography
LC–MS: Liquid chromatography mass spectrometry
mMRC: Modified British medical research council
MS: Combined with mass spectrometry
OPLS-DA: Orthogonal partial least squares-discriminant analysis
PCA: Principal component analysis
PLS-DA: Partial least squares discriminant analysis
QC: Quality control
ROC: Receiver operating characteristic
RSD: Relative standard derivation
